# Reversal of Liver Fibrosis

**DOI:** 10.4103/1319-3767.45072

**Published:** 2009-01

**Authors:** Mona H. Ismail, Massimo Pinzani

**Affiliations:** Department of Internal Medicine, Division of Gastroenterology at King Fahad Hospital of the University, Al-Khobar, Saudi Arabia; 1 Dipartimento di Medicina Interna-Center for Research, High Education and Transfer “DENOThe”- Università degli Studi di Firenze, Florence, Italy

**Keywords:** Antifibrotic, cirrhosis, fibrogenesis, liver fibrosis

## Abstract

Hepatic fibrosis is a scarring process associated with an increased and altered deposition of extracellular matrix in the liver. It is caused by a variety of stimuli and if fibrosis continues unopposed, it would progress to cirrhosis which poses a significant health problem worldwide. At the cellular and molecular level, this progressive process is characterized by cellular activation of hepatic stellate cells and aberrant activity of transforming growth factor-β with its downstream cellular mediators. Liver biopsy has been the reference test for assessment of hepatic fibrosis, but because of its limitations, noninvasive markers of liver fibrosis were developed. Liver fibrosis or cirrhosis was considered irreversible in the past but progress of research on the molecular pathogenesis of liver fibrosis has shown that hepatic cellular recovery is possible. Currently, no acceptable therapeutic strategies exist, other than removal of the fibrogenic stimulus, to treat this potentially devastating disease.

Liver fibrosis, or scarring, occurs as an attempt to limit tissue damage in response to chronic liver injury, regardless of the etiology. It is characterized by excessive deposition of extracellular matrix (ECM) proteins, especially alpha 1 collagen[1] and is associated with major alterations in the quantity and composition of ECM. Liver fibrosis is initiated by a cascade of events resulting in hepatocyte damage, recruitment of inflammatory cells to the injured liver, and activation of collagen-producing cells. Hepatic stellate cells (HSC) are a major source of collagen type 1.[2] The fibrogenic response is a complex process in which accumulation of ECM proteins, tissue contraction, and alteration in blood flow are prominent. Progressive scarring in response to a persisting liver insult eventually results in cirrhosis which is one of the leading causes of death worldwide and a major global health burden. Liver fibrosis was considered an irreversible process in the past, however, recent advances and understanding of hepatic cellular processes and molecular biology have resulted in accumulation of clinical and experimental evidence of hepatic cellular recovery with possible remodeling of scar tissue.[3]

## DEFINITION OF FIBROSIS AND CIRRHOSIS

Liver fibrosis results from perpetuation of the normal wound healing response, resulting in an abnormal continuation of fibrogenesis. It is characterized by an excessive deposition of ECM proteins which includes three large families of proteins – glycoproteins, collagens, and proteoglycans.[4] Fibrosis occurs as a result of repeated cycles of hepatocytes injury and repair. The cascade of events that establish hepatic fibrosis is complex, and is influenced by how different cell types in the liver interact in response to injury, and activation of HSC is the central event.[5] Liver fibrosis is a dynamic process; it is usually secondary to hepatic injury and inflammation, and progresses at different rates depending on the etiology of liver disease and is also influenced by environmental and genetic factors.[6,7] If fibrosis continues unopposed, it would disrupt the normal architecture of the liver which alters the normal function of the organ, ultimately leading to pathophysiological damage of the liver. Cirrhosis represents the final stages of fibrosis.[8] It is characterized by fibrous septa which divide the parenchyma into regenerative nodules[9] which leads to vascular modifications and portal hypertension with its complications of variceal bleeding, hepatic encephalopathy, ascites, and hepatorenal syndrome. In addition, this condition is largely associated with hepatocellular carcinoma with a further increase in the relative mortality rate.[10]

## PATHOGENESIS OF LIVER FIBROSIS

The mechanisms able to elicit and sustain liver fibrogenesis may be classified in three main groups: a) chronic activation of the wound healing reaction, b) oxidative stress, and c) Derangement of epithelial–mesenchymal interactions and epithelial–mesenchymal transition in cholangiopathies.

### Chronic activation of the wound healing reaction

Similar to what was observed in other fibrogenic disorders affecting different organs and systems, the chronic activation of the wound-healing reaction is the most common and relevant mechanism in hepatic fibrogenesis. Overall, hepatic fibrogenesis due to the chronic activation of the wound healing reaction is characterized by the following key features: i) the persistence of hepatocellular/cholangiocellular damage with variable degree of necrosis and apoptosis; ii) a complex inflammatory infiltrate including mononuclear cells and cells of the immune system; iii) the activation of different types of ECM-producing cells (HSCs, portal myofibroblasts (MFs), etc.) with marked proliferative, synthetic, and contractile features; iv) marked changes in the quality and quantity of the hepatic ECM associated with very limited or absent possibilities of remodeling in the presence of a persistent attempt of hepatic regeneration.[11] Work performed in the past two decades have highlighted the role of several growth factors and cytokines involved in the chronic wound healing reaction and affecting the profibrogenic potential of HSC. At present, most of the research on soluble factors potentially affecting the development of liver fibrosis is focused on the role of adipokines and their possible involvement in nonalcoholic fatty liver disease (NAFLD) and nonalcoholic steatohepatitis (NASH).[12] A possible interplay and/or association between fibrogenesis and angiogenesis in chronic liver disease (CLD) is suggested and supported by several findings: angiogenesis and upregulation of vascular endothelial growth factor (VEGF) expression has been documented in different models of acute and chronic liver injury as well as in specimens from human fibrotic/cirrhotic liver and hepatocellular carcinoma.[13,14] From a mechanistic point of view, angiogenesis in fibrogenic CLD can be interpreted according to two main pathways. First, the process of chronic wound healing, typical of fibrogenic CLD, is characterized by an overexpression of several growth factors, cytokines, and metalloproteinases (MMPs) with an inherent proangiogenic action.[15] Second, neoangiogenesis is stimulated in hepatic tissue by the progressive increase of tissue hypoxia. This mechanism is strictly linked to the anatomical modifications following the establishment of periportal fibrosis with an increased contribution of the hepatic artery to the formation of sinusoidal blood. Accordingly, sinusoidal blood flow becomes increasingly arterialized with hepatocytes adjusting to an abnormally high oxygen concentration. Subsequently, the progressive capillarization of sinusoids leads to an impairment of oxygen diffusion from the sinusoids to hepatocytes with the consequent upregulation of proangiogenic pathways.[16,17] An elegant and convincing demonstration of the interplay among inflammatory response, angiogenesis, and fibrogenesis has been recently provided by an experimental study in which all these features have been significantly reduced by the treatment with the multitargeted receptor tyrosine kinase inhibitor Sunitinib.[18]

### Oxidative stress

Involvement of oxidative stress has been documented in all human major clinical conditions of CLD as well as in most experimental models of liver fibrogenesis,[19] but it is likely to represent the predominant profibrogenic mechanism mainly in NAFLD/NASH and alcoholic steatohepatitis (ASH). Oxidative stress in CLD, resulting from increased generation of reactive oxygen species (ROS) and other reactive intermediates as well as by decreased efficiency of antioxidant defenses, does not represent simply as a potentially toxic consequence of chronic liver injury but actively contributes to excessive tissue remodeling and fibrogenesis. ROS and other reactive mediators such as 4-hydroxynonenal (HNE) can be generated outside MFs, here considered as potential ‘target’ cells, being released either by activated inflammatory cells or deriving from hepatocytes, directly or indirectly, damaged by the specific etiological agent or conditions. Indeed, oxidative stress, presumably by favoring mitochondrial permeability transition, is able to promote hepatocyte death (necrotic and/or apoptotic). In some of clinically relevant conditions, generation of ROS within hepatocytes may represent a consequence of an altered metabolic state (like in NAFLD and NASH) or of ethanol metabolism (as in ASH), with ROS being generated mainly by mitochondrial electron transport chain or through the involvement of selected cytochrome P450 isoforms like cytochrome P2E1 (CYP2E1).[20] Oxidative-stress-related mediators released by damaged or activated neighboring cells can directly affect the behavior of human HSC/MFs: ROS or the reactive aldehyde HNE have been reported to upregulate expression of critical genes related to fibrogenesis including procollagen type I, monocyte chemoattractant protein 1 (MCP-1), and TIMP-1, possibly through activation of a number of critical signal transduction pathways and transcription factors, including activation of c-*jun* N-terminal kinases (JNKs), transcription factor AP-1 (AP-1) and for ROS, nuclear factor- kB (NF-kB).[21,22] In addition to ‘profibrogenic’ extracellular release by neighboring cells, ROS generation within human and rat HSC/MFs has been reported to occur in response to several known profibrogenic mediators, including angiotensin II, platelets derived growth factor (PDGF), and the adipokine leptin.[23] A final concept to mention is the fact that oxidative stress may contribute to CLD progression also by affecting the immune response. Experimental studies (alcohol fed rodents) and clinical data (patients affected by alcoholic liver disease (ALD), chronic hepatitis C virus (HCV) infection or NAFLD) indicate that oxidative stress is associated with the development of circulating IgG antibodies directed against epitopes derived from proteins modified by lipid peroxidation products or against oxidized cardiolipin. Of relevance, titer of these antibodies correlates with disease severity and, as recently proposed for NAFLD patients, may serve as prognostic predictor of progression of NAFLD to advanced fibrosis.[24]

### Derangement of epithelial–mesenchymal interactions and epithelial–mesenchymal transition in cholangiopathies

Cholangiopathies represent a group of progressive disorders and are considered a major cause of chronic cholestasis in adult and pediatric patients. They share a common scenario that involves coexistence of cholestasis, necrotic or apoptotic loss of cholangiocytes, cholangiocyte proliferation, as well as portal/periportal inflammation and fibrosis. The so-called ‘ductular reaction’ (i.e., proliferation of bile ductular cells or cholangiocytes) has been seen as the ‘pace maker of portal fibrosis’; intense proliferation of these epithelial cells is associated with significant changes in the surrounding mesenchymal cells (first portal fibroblasts and then HSCs with parenchyma invasion) and ECM.[25] It has long been unclear whether the first event was represented by phenotypic changes in proliferating cholangiocytes or by changes in ECM leading to epithelial cell proliferation. However, an intense cross-talk between mesenchymal and epithelial (i.e., cholangiocytes) cells has been suggested to underlie the release of cytokines and proinflammatory mediators possibly responsible for the overall cholangiopathies. As a matter of fact, cholangiocytes are now considered as active ‘actors’ in pathological conditions by their ability to secrete chemokines (Interleukin-6 (IL-6), tumor necrosis factor α (TNF β), Interleukin-8 (IL-8), and MCP-1) and profibrogenic factors (Platelets derived growth factor (PDGF-BB), endothelin 1 (ET-1), connective tissue growth factor (CTGF), and transforming growth factor beta 2 (TGF β2). All these factors, which can also be produced by infiltrating immune, inflammatory, or mesenchymal cells, may affect, in turn, both epithelial cells and their intense cross-talk with mesenchymal cells, thus sustaining the fibrogenic response.[26] However, very recently different laboratories are accumulating preliminary evidence suggesting that the scenario of cholangiopathies may be initiated by a process of ‘epithelial-mesenchymal transition’ involving cholangiocytes and possibly driven by TGF β.[27]

## DIAGNOSIS OF FIBROSIS

The complete evaluation of a patient with diffuse liver diseases requires clinical evaluation, laboratory tests, and pathological examination. The liver biopsy is regarded as the historical ‘gold standard’ for diagnosis and assessment of prognosis in CLD.[28,29] At least three scoring methods are commonly used to stage liver fibrosis: the Knodell, Ishak, and METAVIR scores.[30,31] The Knodell and METAVIR score fibrosis from stage 0–4, with stage 4 as cirrhosis, whereas Ishak scores fibrosis from 0–6 where 5 is incomplete or early cirrhosis and 6 indicates established cirrhosis.[32] These methods are semi-quantitative and the invasiveness of liver biopsies with its associated life-threatening risks and morbidity make it a poor choice when considering assessment of liver fibrosis progression or regression. Furthermore, there is the issue of sampling error, defined as variable levels of fibrosis throughout the liver, with biopsy only examining a small (1/50,000) portion of the liver.[33,34] Liver biopsy has been shown to have significant inter and intraobserver variability among pathologists, with an average 20% error rate in the staging of fibrosis.[35] The minimum suitable length of liver tissue needed for assessing liver fibrosis reliably is 25 mm and the presence of an experienced hepatopatholgist is important.[34]

Over the past years, several noninvasive tests have become available to assess liver fibrosis, primary in patients with chronic hepatitis C infection.[36,37] The currently available noninvasive tests, which are surrogate markers of liver fibrosis (direct markers of fibrosis), such as serum hyaluronate, Type IV collagen, matrix metalloproteinase 1 (MMP), tissue inhibitor of matrix metalloproteinase-1 (TIMP-1), laminin, and TGF β, have limited accuracy for diagnosis of significant fibrosis (METAVIR ≥ F2 or Ishak >3). Other noninvasive tests (indirect markers of liver fibrosis) include FibroTest-ActiTest,[38] APRI,[39] Forns fibrosis index,[40] and enhanced liver fibrosis (ELF) score.[41,42] The diagnostic performance of these indices is generally good, with a receiver operating characteristics (ROC) curve ranging from 0.77–0.88. Parkes *et al*. conducted a systematic review to assess the performance of panels of serum markers of hepatic fibrosis in chronic HCV infection, incorporating analyses placing markers in a clinical context. They concluded that serum markers can rule-in or rule-out fibrosis in up to 35% of patients, but cannot differentiate stages of fibrosis reliably and improvement of index and reference test is needed.[43]

FT-AT, from Biopredictive, Paris, France, is a noninvasive blood test that combines the quantitative results of six serum biochemical markers (alfa2-macroglobulin, haptoglobin, gamma glutamyl transpeptidase, total bilirubin, apolipoprotein A1, and alanine aminotransferase (ALT)) with patients’ age and gender in a patented algorithm in order to generate a measure of fibrosis and necroinflammatory activity in the liver.[38,44] FT-AT provides an accurate measurement of bridging fibrosis and/or moderate necroinflammatory activity with area under the receiver operating curve (AUROC) predictive value between 0.70 and 0.80, when compared to the liver biopsy.[45,46]

Recently, transient elastography or Fibroscan (Echosens, Paris, France) has become available, which measures liver stiffness or elasticity to assess liver fibrosis.[47] The scan was developed on the principle that livers with increasing degrees of scarring or fibrosis have decreasing elasticity and that a shear wave propagating through stiffer material would progress faster than in one with more elastic material.[48] Transient elastography is painless, rapid, and easily performed at the bedside or in the outpatient clinic. A recent systemic review identified twelve studies, 9 for FibroTest (*N* = 1,679) and 4 for Fibroscan (*N* = 546) and the area under the curve (AUCs) for FibroTest and Fibroscan were 0.90 (95% CI not calculable) and 0.95 (95% CI 0.87–0.99), respectively. The authors concluded that FibroTest and Fibroscan have excellent diagnostic accuracy for the identification of HCV-related cirrhosis, but lesser utility for earlier stages of fibrosis.[49] The combined use of transient elastography and biochemical markers seems to be the most promising noninvasive techniques which can help the clinician decide whether a liver biopsy is necessary in some patients, and accordingly decide who to treat[37] [Table 1]

**Table 1:** Common noninvasive tests of liver fibrosis

Test	Parameters	Patients	AUC	PPV/NPV (%)
Fibrotest[38] 2001	α2-macroglobulin, hepatoglobulin, lipoprotein	HCV	0.83	>90/100
	A1, bilirubin and δ-glubulin.			
Forns fibrosis index[40] 2002	Age, platelet count, GGTP and cholesterol.	HCV	0.86	66/96
APRI index[39] 2003	AST/Platelet Ratio	HCV	0.80	Fibrosis: 91/90
				Cirrhosis: 65/100
ELF score[41] 2004	MMP-3, TIMP1	Mixed CLD	0.80	90/92

GGT: g-glutamyl-transpeptidase, AST: aspartate transaminase, TIMP-1: tissue inhibitor of matrix metalloproteinase 1, MMP-3: matrixmetalloproteinase-3, CLD: chronic liver disease, HCV: hepatitis C virus

## REVERSAL OF FIBROSIS OR CIRRHOSIS

The concept of reversibility of liver fibrosis and cirrhosis is not new. Popper and Udenfreind emphasized the importance of enzymatic processes to fibrosis regression in 1970.[50] Perez-Tamayo in 1979 wrote a review entitled “Cirrhosis of the liver: a reversible disease?” and enumerated evidence for reversibility of fibrosis and cirrhosis in both animal models and human disease provided the inciting agent is discontinued and sufficient time is allowed for the injured liver to recover.[51] Fibrosis usually requires at least several months to years of ongoing insult. However, not all patients exposed to a similar causal agent develop the same degree of liver fibrosis, that is, patients with similar risk factors have some variability in progression of liver fibrosis, which also may reflect host genotypic polymorphisms.[7] Evidence of fibrotic regression has now been documented in the entire spectrum of CLDs, including autoimmune hepatitis, biliary obstruction, iron overload, NASH, and viral hepatitis B and C.[52–58]

The issue of regression/reversibility of cirrhosis originates from evidence obtained in animal models upon the discontinuation of the cause of liver damage or following treatment with a putative antifibrotic agent. Although a regression has been shown in animal models of cirrhosis this possibility is not yet fully substantiated in humans. Evidence of either fibrotic or cirrhotic regression has now been reported in CLD of different etiologies, including viral hepatitis,[59–65] autoimmune hepatitis,[52] alcoholic, and nonalcoholic steatohepatitis.[55,66,67] However, when performing an accurate analysis of the results of these studies, the only prudent conclusion is that, in most cases, there was a variable degree of fibrosis regression in cirrhosis but not a reversal of cirrhosis.[25,68] Along these lines, there is no convincing evidence that the abnormalities of the intrahepatic vasculature regress in human cirrhotic liver. Actually, the available evidence suggests that the so-called veno-portal adhesions persist even in cases of extensive fibrosis regression, and evident ‘arterialized’ sinusoids appear in the context of intrahepatic arterio-venous shunts.[69] The most obvious problem when discussing the issue of fibrosis regression in cirrhosis or even cirrhosis reversal is the lack of a clear and common language. Ultimately, what we need is a precise distinction of advanced fibrosis (‘precirrhosis’) from true cirrhosis and the possibility of staging cirrhosis. The problem is fundamentally based on the use of semi-quantitative scoring systems for staging fibrosis and, in particular, the fact that cirrhosis is always represented by the highest score and is indeed considered as an end-stage of CLD.[25,70] In fact, cirrhosis appears in a very broad spectrum of variants (early, fully developed, ‘active’, and ‘inactive’) and more than one study has documented the transition from micronodular to macronodular cirrhosis following the discontinuation of the causative agent.[71,72] Practically, as clearly stated by Desmet and Roskams,[25] there is a fundamental difference between a diagnosis of cirrhosis and a score of cirrhosis. For example, a low score does not exclude cirrhosis of the macronodular or incomplete septal type, which can be due to sampling error. From a clinical point of view, patients with cirrhosis can experience a widely variable clinical course and the cirrhotic stage defined as “compensated cirrhosis” includes anything from the initial histopathological demonstration of ‘early cirrhosis’ to the development of complications of portal hypertension. This oversight is mainly motivated by the fact that, until recently, it hardly mattered whether a patient had ‘early’ or ‘late’ cirrhosis since the only viable option was liver transplantation, and it is clearly reflected by clinical staging systems such as the mayo end stage liver disease (MELD) score.[73]

The increasing clinical awareness that cirrhosis represents a new dimension in the clinical course of CLD and not just the extreme stage of fibrosis, together with the more and more realistic possibility of reducing fibrosis even in a cirrhotic liver have led to the assumption that liver transplantation is no longer the only possible option to increase patient survival. Importantly, according to epidemiological data concerning two of the most common CLDs, i.e., HCV and NASH, and the relative estimates of disease progression, the number of patients with definite cirrhosis will increase exponentially in the next 10–15 years, thus representing the most frequent clinical entity in hepatology practice.[74,75]

The possibility of monitoring fibrosis regression in cirrhosis faces the already mentioned lack of a system able to classify cirrhosis in different stages. A distinction should be made between ‘compensated’ (i.e., complication-free) and ‘decompensated’ (i.e., with clinically evident complications of portal hypertension) cirrhosis. In this context, a classification of compensated cirrhosis represents the major clinical need when analyzing the effect of a causative and/or antifibrotic therapy aimed at prolonging complication-free survival. Different approaches have been proposed in order to reach this goal. First, as suggested by Goodman *et al.*[76] morphometric image analysis may help quantify the extension of fibrosis in cirrhotic liver, thus overcoming the biases of the semi-quantitative scores and to provide staging of cirrhosis beyond an end-stage score. However, morphometric analysis of hepatic collagen content may be also limited by potential sampling variability and is not reliable when dealing with fragmented specimens. Second, the measurement of hepatic venous pressure gradient (HVPG), a validated, safe, and highly reproducible technique, has been proposed for monitoring the progression of the disease from the precirrhotic to advanced stages of cirrhosis.[77] In absence of significant fibrotic evolution, HVPG, as an expression of intrahepatic resistance, does not exceed 5 mm Hg, whereas a gradient of more than 5 mm Hg is always associated with significant fibrosis. Cross-sectional studies have shown that portal pressure (estimated by the HVPG) must reach certain thresholds for the development of complications of portal hypertension: 10 mm Hg for the development of varices and ascites (‘clinically significant’ portal hypertension) and 12 mm Hg for variceal bleeding (‘clinically severe’ portal hypertension).[78] Therefore, in broad terms, at least cirrhotic patients with a HVPG within the range 5–10 mm Hg should be complication-free, i.e., ‘compensated’. Third, the majority of the so far proposed noninvasive methodologies for the evaluation of fibrosis progression in CLD, including serum biomarkers and transient elastography,[79] seem to have an adequate diagnostic accuracy for the prediction of advanced fibrosis and cirrhosis and could be further implemented for staging cirrhosis. Along these lines, a 3-variable algorithm consisting of hyaluronic acid, TIMP-1, and platelet count was recently shown to correlate with histopathological scores better than with the hepatic collagen content measured by morphometric analysis.[80] In addition, in patients with compensated HCV cirrhosis, with a HVPG in the range 5–12 mm Hg, liver stiffness measurement (LSM) by transient elastography shows an excellent correlation with HVPG values and may be a good predictor of significant and severe portal hypertension.[81] Therefore, it is imaginable that the use of noninvasive methodologies could represent a feasible way to complement traditional/morphometric histopathological analysis and the measurement of HVPG in the attempt of staging compensated cirrhosis and assessing response to treatment.

## LIVER FIBROSIS AND ANTIFIBROTIC THERAPY

Advances in understanding of the pathophysiologic basis of fibrogenesis are now leading to novel therapeutic approaches.[82,83] Cure of the primary disease to prevent ongoing injury remains the most effective strategy to reverse fibrosis. Existing treatments, particularly those that treat the primary injury, can allow complete resolution. Although it is likely that newly synthesized collagen may be more susceptible to degradation than old collagen, there is abundant evidence in animal models that even advanced cirrhosis is reversible, and in humans, the data suggest that fibrosis is reversible.[52,53,62,84,85] Antifibrotic therapies would target different areas in the fibrogenic cascade, including inhibition of matrix deposition, collagen synthesis, modulation of stellate cell activation, enhancing matrix degradation, or stimulation of stellate cell death or apoptosis. Several drugs with specific ‘antifibrotic activity’ have been studied in human trials but were not proved to be clearly effective. The ideal antifibrotic agent which is safe, when used over a long time, specific to the liver and nontoxic to hepatocytes, potent, orally bioavailable, and inexpensive is not yet available. Many agents were shown to be effective *in vitro* and in animal models. Translation of this laboratory success into clinical trials is underway, paving the way for use in human liver disease.[86] Increasingly, multiple-agent strategies that work at different mechanistic levels are likely to be assessed (combination therapy) [Table 2]. Evidence of the long-term benefits of the reversal of fibrosis on clinical outcome, such as a reduction in portal hypertension or the rate of development of hepatocellular carcinoma, is needed.[87] Although there are no definite and effective antifibrogenic agents, possible candidates are antioxidants,[88,89] interferons,[90] flavonoids,[91] renin-angiotensin system inhibitors,[92–94] endothelin receptor antagonists,[95] and peroxisome proliferator activated receptor-gamma (PPAR-gamma) agonists.[96,97]

**Table 2:** Antifibrotic agents

Agent	Disease	Activity
α-Tocopherol	HCV and others	Downregulation of collagen type 1 and αSMA. Inhibit HSC activation.
Interferon-δ	HBV and HCV	Inhibit ECM synthesis in HSC.
Quercetin: a flavonoid	CCL4 in rats	Antioxidant and free radical-scavenging
ACE inhibitor	CLD	Inhibit HSC proliferation.
PPAR-δ Agonist	NASH	Reduction in α1 procollagen, α-SMA and MCP-1 and upregulation of MMP-3.

ACE: Angiotensin converting enzyme, CLD: chronic liver disease; HCV: hepatitis C virus, HBV: hepatitis B virus, MCP-1: Monocyte chemoattractant protein 1, MMP-3: matrixmetalloproteinase-3, HSC: Hepatic stellate cell, ECM: Extracellular matrix, CCL4: Carbon tetrachloride, α-SMA: alpha smooth muscle actin

## CONCLUSIONS

Reversal of fibrosis is a reality and treatment of the primary cause of injury can allow complete resolution of fibrosis. Noninvasive approaches in diagnosis of fibrosis are still evolving but promising. Our understanding of the mechanism of liver fibrosis has changed dramatically over the last decade and is no longer viewed as either passive or permanent but as a dynamic process. Many mechanisms and potential therapies continue to be identified and more research is required. The number of potential therapeutic targets has exploded in recent years and the realization that fibrosis can regress lends new urgency to their investigation.
